# Transfer of Bisphenol A and Trace Metals from Plastic Packaging to Mineral Water in Ouagadougou, Burkina Faso

**DOI:** 10.3390/ijerph20206908

**Published:** 2023-10-11

**Authors:** Boukary Sawadogo, Francis Ousmane Konaté, Yacouba Konaté, Ousmane Traoré, Seyram Kossi Sossou, Eric Sawadogo, Pane Bernadette Sourabié Ouattara, Harouna Karambiri

**Affiliations:** 1 Laboratoire Eaux Hydro-Systèmes et Agriculture (LEHSA), Institut International D’Ingénierie de l’Eau et de l’Environnement (2iE), 1 Rue de la Science, Ouagadougou 01 BP 594, Burkina Faso; yacouba.konate@2ie-edu.org (Y.K.); seyram.sossou@2ie-edu.org (S.K.S.); eric.sawadogo2ie@gmail.com (E.S.); harouna.karambiri@2ie-edu.org (H.K.); 2 Agence Nationale Pour la Sécurité Sanitaire de L’Environnement, de L’Alimentation, du Travail et des Produits de Santé (ANSSEAT), Boulevard des Tensoba, Ouagadougou 09 BP 24, Burkina Faso; konatefrancis@yahoo.fr (F.O.K.); us_traore@yahoo.fr (O.T.); b_sourabie@yahoo.fr (P.B.S.O.)

**Keywords:** Bisphenol A, drinking water quality, plastic containers, sachets of mineral water, Sudano-Sahelian climatic conditions, trace metals, water micropollutants

## Abstract

The consumption of packaged water is growing rapidly in both urban and rural centres in Burkina Faso. Bisphenol A (BPA) and trace metals are among the compounds used in the manufacture of plastic packaging, and their presence in water can pose a health risk to consumers due to their alleged toxicity. Therefore, this study explores the transfer of these compounds from plastic packaging to mineral water in Sudano-Sahelian climatic conditions. Ten samples of packaged sachet water commercialised in Ouagadougou were studied. An absence of BPA in the borehole water used to produce packaged water has been shown. The transfer of BPA into mineral water increases with storage temperature. The BPA that appears in packaged water degrades over time. BPA concentrations ranged from 0 to 0.38 mg/L after two weeks of storage, 0 to 0.8 mg/L after four weeks of storage and 0 to 0.35 mg/L after 8 weeks of storage. Analysis of the trace metals showed steadily increasing concentrations from the second to the sixth weeks, with concentrations ranging from 0 to 9.7 µg/L for cadmium and from 0 to 0.13 mg/L for iron in the sachet water samples.

## 1. Introduction

Africa is currently characterised by rapid demographic growth, with one of the highest birth rates in the world [1]. The increasingly numerous population is become urbanised, with an average urbanisation rate of 4% [2]. The urban population growth dynamic has risen over the years from 31.0% in 1990 to 54.4% in 2020, while in West Africa, the growth rate has increased from 7.5% to over 34.0% from 1960 to 2020. This translates to more than 123 million inhabitants in West African cities in 2020 [3]. This new environment is bringing about profound changes in lifestyle and consumption patterns. Feeding and maintaining this urban population calls for intensification of production. Therefore, the situation places significant pressure on available resources, especially on the limited water resources. Burkina Faso, just like other countries in West Africa, faces major challenges in access to water and sanitation amidst the galloping population growth. Urbanisation in Ouagadougou remains heterogeneous [4]. Against this backdrop of urbanisation and the soaring water demand, it has been observed that people are increasingly using packaged water as drinking water, both at home and in the workplace [5]. Packaged water is produced by local industrial units. The hygienic appearance of these waters appeases consumer confidence. The situation has thus promoted the creation of numerous packaged water production units.

However, the rush to create packaged water production units must not compromise consumer protection, as failure to comply with specific requirements can lead to products that do not adhere to the potability standards in force for drinking water, especially as water is a mass consumption product. This is why quality control, through regular monitoring of the water produced, is an important part of the process.

Most water quality monitoring protocols focus on common parameters that can immediately impact consumer health. However, it is recognised that water can be contaminated in numerous ways [6,7,8,9]. There is natural contamination, i.e., depending on the composition of the rocks making up the aquifer [10,11,12,13], and/or anthropic contamination due to human activities [14]. The latter is complex because it varies according to human activities and different uses of the resource [15,16,17]. Some water pollutants can be toxic through a cumulative effect if ingested repeatedly. This is particularly true of certain micropollutants such as trace metals and bisphenol A (BPA) [18,19]. BPA, whose chemical name is 2,2-bis(4-hydroxyphenol) propane, is a synthetic chemical mainly used to manufacture plastics and resins. It is obtained through a reaction between two phenol equivalents and one acetone equivalent, with its molecule containing two phenol functional groups [20,21]. Mineral water is generally packaged in plastic containers produced with BPA [22]. Under normal conditions, BPA is a chemically stable substance, but it breaks down slowly at high temperatures and is released into the environment [23,24]. BPA is considered to be an endocrine disruptor [22,25]. It is also considered by several authors to be the cause of numerous diseases [19,26,27]. In recent years, BPA has been the subject of numerous studies worldwide. Authors have been particularly interested in its presence in water and its toxicity for humans and the environment [28,29]. Other studies have often focused on its transfer from plastic bottles to water or from cans to food [30,31]. Trace metals are also associated with various risks for humans in the event of high levels in drinking water [32,33,34]. The novelty of this study deals with the transfer of BPA and trace metals from plastic packaging to packaged mineral water; we assess their levels in packaged water being produced and commercialised in an urban centre in Africa. The external and internal factors involved in their migration from plastics to packaged water were studied.

## 2. Materials and Methods

### 2.1. Study Area

**Figure 1:**
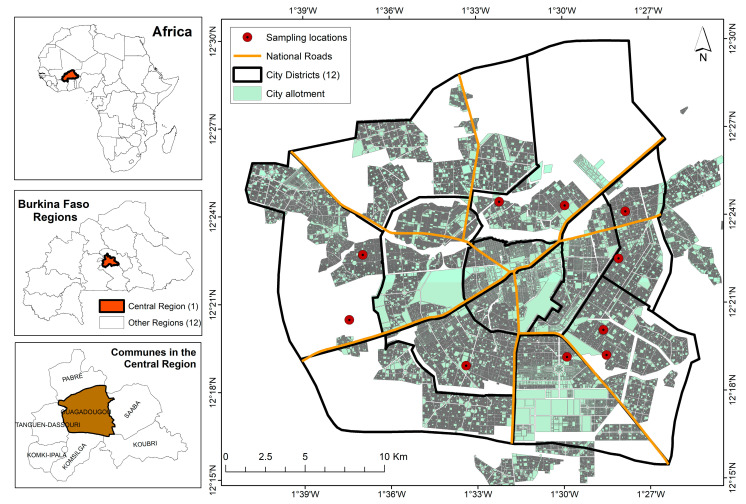
Location of the city of Ouagadougou.

The study was carried out in Ouagadougou, the capital city of Burkina Faso. It is the country’s largest economic and cultural centre, with an estimated population of two million six hundred and eighty-four thousand and fifty-two (2,684,052) inhabitants in 2020 [35]. The city is growing steadily, with an annual growth rate of 7.6% [5]. Ouagadougou is the capital of the Centre region, with twelve (12) districts subdivided into fifty-two (52) sectors. The city has a surface area of 2857.124 km^2^ and is located between parallels 12°30′ and 12°25′ north latitude and meridians 1°27 and 1°35 west longitude. Figure 1 shows a map of the city of Ouagadougou.

### 2.2. Sampling and Preliminary Tests

**Figure 2:**
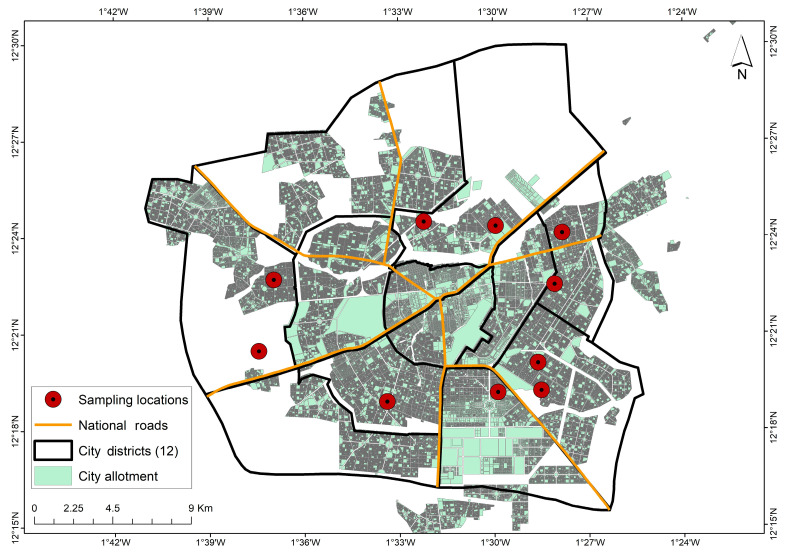
Geographical distribution of samples in the city of Ouagadougou.

The National Agency for Environmental, Food, Labour, and Health Product Safety in Burkina Faso (Ansseat) monitors water consumed in the city of Ouagadougou. Units that produce mineral water sign an assistance contract with this agency, allowing it to monitor their water quality. A sample of ten brands of mineral water packaged in sachets were selected from among the units that had signed a service contract with Ansseat for the study. In order to gain an overview of the transfer of these substances, the samples were selected based on the physical nature of the containers. Packaging from different water production units differs according to the presence or absence of a writing tab, as well as the roughness and thickness of the sachet. Other criteria used to select the samples were the city’s geographical distribution of the production units. Table 1 presents the physical characteristics of the sachets while the geographical location of the samples in the city is shown in Figure 2. The final criterion for sample selection was biodegradability; the test was carried out using an XRF Tester as described by Hagiwara et al. [36]. The plastic containers were scanned to determine their iron, manganese, and cobalt concentrations and their biodegradability. Many metals such as iron, lead, cadmium, cobalt, manganese, antimony, and many other trace elements are used in the composition of plastics to improve their quality and accelerate their biodegradability. This therefore informed the selection of containers with high levels of these metals to determine whether they are transferred to the packaged water. Table 2 shows the iron, manganese, and cobalt contents given by the XRF tester, used as criteria for choosing our samples. Considering all the selectivity criteria, ten (10) samples were selected for the rest of the study (Table 1). For each production unit, samples were taken from a pack of forty sachets of packaged water (W1 to W10). The borehole water used to produce the sachet water (W1 to W10) are named F1 to F10, respectively. The quality of the borehole water was analysed.

### 2.3. Analyses and Calibration

BPA was analysed using an Agilent gas chromatograph-mass spectrometer (GC-MS) [31,37,38,39]. The analyses were carried out after an internal validation of the method. Before carrying out the analysis of BPA in the various samples, the method was verified. The purpose of this verification was to determine the effectiveness or efficiency of the method prior to its use in this study. A calibration range was prepared using standard solutions. Eight solutions of different concentrations (12.50 mg/L, 6.25 mg/L, 3.13 mg/L, 1.56 mg/L, 0.95 mg/L, 0.39 mg/L, 0.10 mg/L, and 0.05 mg/L) of BPA were prepared for GC-MS calibration using acetonitrile and ultrapure water as a solvent. The sample was spiked to verify the method’s performance using the method described by Errico et al. [40]. Dichloromethane is the solvent used for PBA extraction. Table 3 shows the recovery rates for Bisphenol A using GC/MS as a function of the concentrations previously spiked. The internal procedure recommends a recovery rate of between 75% and 120%.

Once the method had been successfully tested, all the samples were treated in accordance with the method using the GC-MS. For each sample, the groundwater was analysed at ambient temperature. To assess the effect of temperature, the packaged water samples were placed in ovens set at 18 ± 2 °C, 29 ± 2 °C, and 39 ± 2 °C. The duration of conditioning varied from two, four, and eight weeks to study the combined effect of temperature and storage time. This was performed to monitor the behaviour of BPA and trace metals contained in the plastic container. These three sample conditioning temperatures were given by the meteorological surveys of the city of Ouagadougou:

18 °C as the average of the lowest annual temperatures;
29 °C as the average annual temperature;
39 °C as the average of the highest annual temperatures.

Samples collected from the boreholes were immediately acidified with nitric acid at a concentration around 5% *v*/*v*. Trace elements were measured with an Avio Perkin Elmer inductively coupled plasma atomic emission spectrophotometer (ICP-OES). The direct method was used to assay the samples. The calibration range was carried out using a multi-element standard.

## 3. Results and Discussion

### 3.1. Production, Transport and Storage of Packaged Water

The production and marketing of packaged water is an activity involving a chain of operations. A better interpretation of the results requires an in-depth examination of the various elements in the chain. Similarly, as several parameters can influence the fate of BPA and trace metals, the impact of transport and storage conditions on transfer to the product was highlighted.

Although all the production units use borehole water, the water circuit from the borehole to the bagging unit differs from one unit to another. The most complex systems consist of pre-treatment and demineralisation devices with units based on dense membranes and tanks for adding mineral substances, a contrast to simple circuits with water distribution from a storage tank. Based on the analysis, the semi-automated units seem to offer a better guarantee of the final product’s quality and its compliance with good production standards than products from the smaller units.

After production, the finished product must be transported to wholesale distributors’ warehouses or retailers’ shelves, i.e., distribution and/or consumer centres. This stage of the production chain involves a variety of means of transport, including lorries, commercial vehicles, and tricycles. Over 60% of products are transported by tricycles because of their relatively low acquisition, servicing, and maintenance costs compared to commercial vehicles and lorries (insurance, technical inspection, motor vehicle tax). These vehicles offer greater flexibility of use and placement in a city where traffic is dense at certain times of the day (driving licence, ability to fill small orders quickly). In addition, more than 65% of means of transport are not covered, which exposes the conditioned water to dust and sunlight.

Storage occurs at three levels: after production, at the sale point, and at the consumer’s home. Before transport, storage is carried out within the water production units. The observations show that the water samples produced are assembled on the floor or on pallets. The findings show that all the production units surveyed had suitable storage conditions. Storage is also carried out at the distribution point. At this level, wholesalers have warehouses in which products are stored. At these points, water packs are generally stored in metal grids provided by the producers. Even when fitted out, these racks often leave the products exposed to the sun, rain, and dust. Therefore, there is a clear risk of contamination and deterioration of water quality at the retail level. At the household level and in retail outlets, storage is relatively better. Consumers use water for direct consumption or storage in coolers, refrigerators, or equipped areas of the house.

Water is therefore stored under different conditions depending on the stage in the consumption chain. Storage at wholesalers, therefore, appears to be the weakest link in the chain, with conditions that do not guarantee product quality. More than half of the packaged water samples were stored in inadequate conditions. Exposure to the sun, wind, dust, and other adverse weather conditions poses the risk of deterioration in product quality. Previous studies have reported that photo-oxidation may be one of the degradation pathways for BPA [41]. It has also been shown that storage conditions do not depend on the brand of the product. It is more generally linked to the distribution company.

### 3.2. Bisphenol A in Sources and Packaged Water in Ouagadougou

The presence of bisphenol A in drinking water can have several origins, the greatest risk of which appears to be transfer from the container to the contents. To a lesser extent, the environmental conditions at the source and a lack of hygiene in handling may also be involved.

The presence of BPA in borehole water was assessed for all ten sources used to produce packaged water. The results of the analyses showed the absence of BPA in all the sampled groundwater used for the production, thus highlighting the fact that the level of BPA pollution in the city of Ouagadougou is low and cannot lead to contamination of the water table. This is justified by the Environment/Health report [42], which shows that the movement of BPA in the water and/or soil matrix is influenced by the physico-chemistry of the receiving soil and the water flowing through it, in particular the pH and the properties of the organic matter in the water. On the other hand, other results reported by Rudel et al. [43] showing concentrations of 1.41 µg/L in groundwater samples taken between a wastewater treatment plant and a municipal landfill, justify that the environmental state of the site on which the production unit is to be located must be closely scrutinised during the health inspection prior to the authorisation to open.

### 3.3. BPA in Packaged Water

#### 3.3.1. Effect of Temperature on BPA Migration

**Figure 3:**
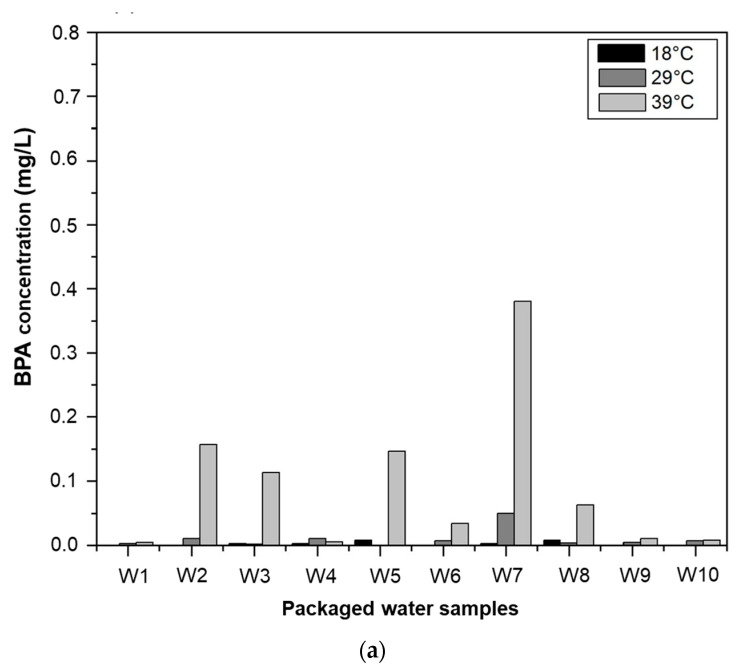

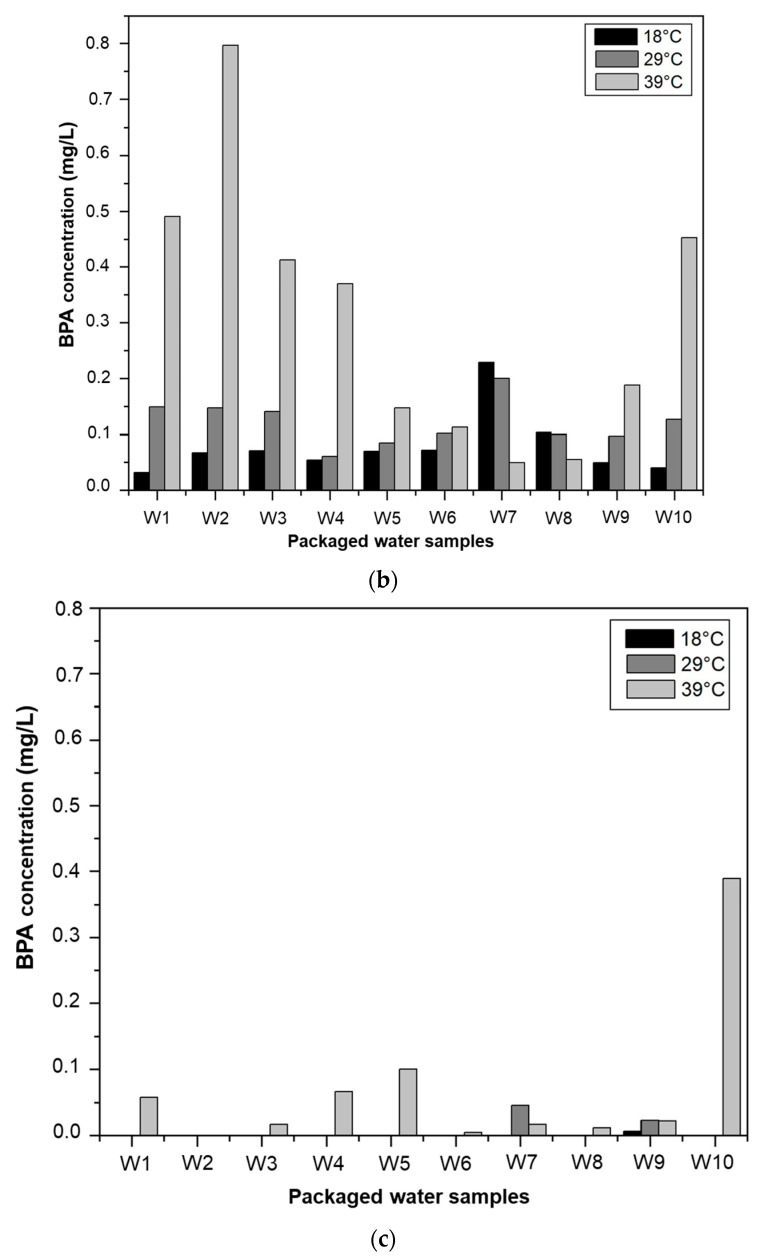
BPA in conditioned water as a function of conditioning time and temperature. (**a**): 2 weeks; (**b**): 4 weeks; (**c**): 8 weeks.

Surveys and observations revealed poor storage conditions for packaged water, particularly regarding exposure to sunlight. As certain studies had revealed the possible effects of temperature on the stability of packaged water, investigations were carried out on the samples taken. The ten samples were therefore subjected to three different temperatures: 18 °C, 29 °C, and 39 °C. Samples were conditioned in ovens at these three temperatures, and the results of the analyses are shown in Figure 3. The results obtained show that although BPA was not present in the water leaving the production lines, BPA levels of up to 0.780 mg/L were obtained in the same packaged water exposed to high temperatures. This substance, previously absent in borehole water, was found in packaged water, suggesting that the containers were the source of contamination. This shows that the sachets used for packaging are made from polycarbonates and/or the resin is made from bisphenol A. These results are in line with those of Cadi et al. [44] who maintain that most plastics are made from polycarbonate or resin, and therefore contain bisphenol A.

It also appears that, at low temperatures, BPA is virtually absent from all the packaged waters studied after 14 days’ storage. The results also show the presence of Bisphenol A in all the samples studied at ambient and high temperatures in the city of Ouagadougou. Analysis of the results shows that the concentration of BPA in packaged water increases with temperature. Thus, a rise in temperature leads to an increase in BPA concentrations in the samples. Temperature therefore accelerates the fragmentation and depolymerization of polycarbonates and peroxide resins, leading to the release of monomers, including BPA [44]. These were also the results of the study by Brede et al. [45], who reported that the use of plastic containers leads to the migration of BPA in water, especially when the temperature is raised. For other authors, such as Calafat et al. [46], the concentration of BPA in water contained in plastic can increase up to 55 times compared to low temperatures. In this 4-week conditioning analysis, BPA concentrations ranged from a minimum of 0.032 mg/L to a maximum of 0.800 mg/L (Figure 3). Taking this maximum concentration into account, subjects weighing 10 kg (average weight of children aged 0–3 years) and 63 kg (average weight of adults) drinking 0.75 L and 1.5 L of sachet water per day, respectively, would have accumulated concentrations of 0.06 and 0.02 mg/kg body weight/day of BPA. Under these conditions of exposure, adults absorb a low dose compared to the acceptable daily intake (ADI), which is 0.05 mg/kg of body weight per day. If packaged water is their only source of BPA contamination, they would be safe. A child weighing 10 kg absorbs 0.06 mg/kg/day, which is higher than the ADI. Although it may not the only source of BPA contamination, the consumption of packaged water leads infants to absorb more than the TDI (0.05 mg/kg of body weight per day). This shows that children are the most exposed to BPA absorption, confirming the thesis of previous authors who maintain that young people in general, and foetuses and newborns in particular, are vulnerable to BPA [47].

#### 3.3.2. Effect of Storage Time on BPA Migration

Figure 3 also shows changes in BPA levels as a function of time. Concentrations at two weeks (Figure 3a) are lower than those at four weeks (Figure 3b). Therefore, the transfer of BPA into the water studied is influenced by the storage time. BPA concentrations in the same sample increased as storage time increased. This indicates that depolymerization does not occur abruptly, but rather as a function of time. This result is in line with those of Sajiki and Yonekubo [48], who showed that the concentration of BPA increases up until day 35. Yoshida et al. [49] reported that storage time is an important factor in BPA migration in products packaged or preserved in plastic containers. A comparison of the results at the two storage times shows different changes in the BPA content in the packaged water samples studied. The transfer of BPA from the sachet to the packaged water is not linear over time. This could be due to the simultaneous degradation of the sachet by physico-chemical and microbiological factors, which contribute to the release of BPA [50,51,52]. On the other hand, the tab of the sachet appears to have no significant impact on the transfer of BPA into the water, whatever the temperature and/or packaging time.

#### 3.3.3. The Degradation of BPA in Packaged Water

**Figure 4:**
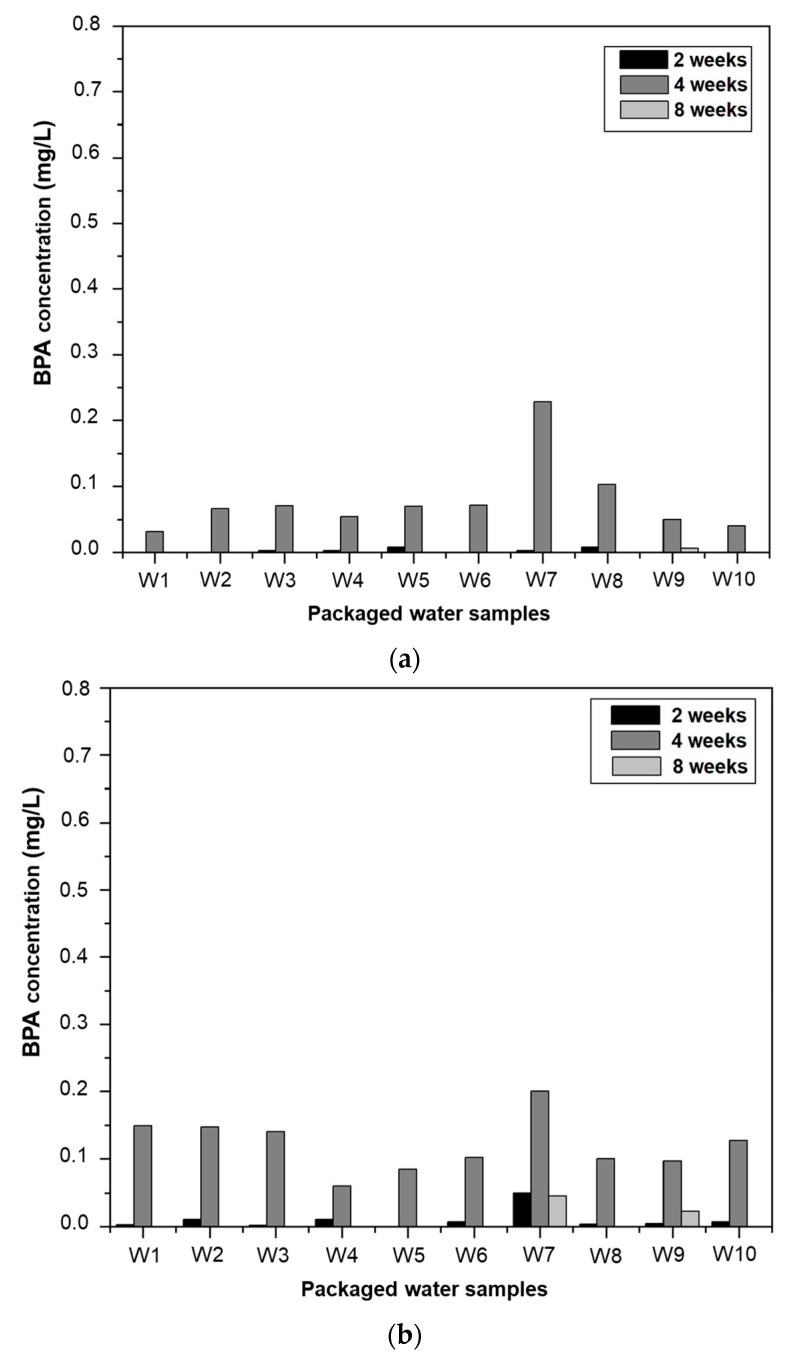

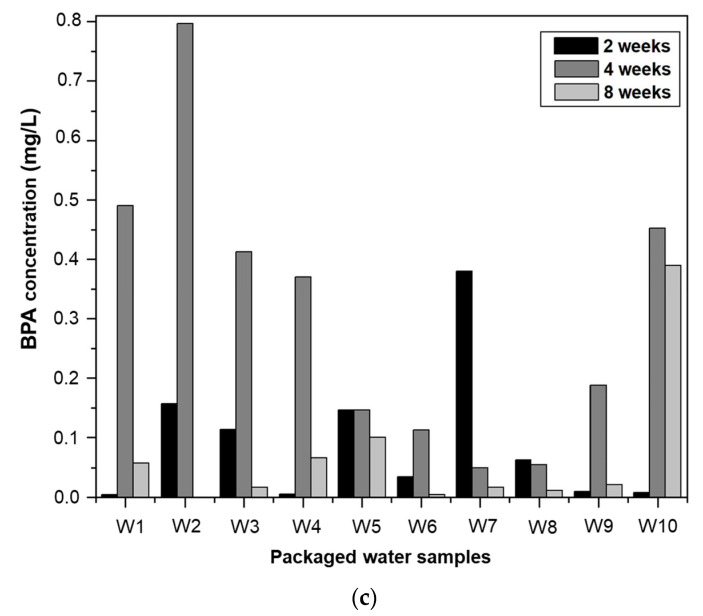
Evolution of BPA in conditioned water as a function of conditioning time and temperature. (**a**): 18 °C; (**b**): 19 °C; (**c**): 39 °C.

BPA is not constantly increasing in packaged water. It degrades under certain conditions, and its content decreases with time. Figure 4 shows the regression of BPA concentrations at two, four, and eight weeks of storage. Between four and eight weeks, concentrations decrease as a function of time. This situation can be explained by a degradation or fragmentation of BPA as the exposure time increases. BPA in contact with oxygen oxidises and degrades, especially in the presence of water. Dissolved oxygen is a major player in the degradation of BPA, and is among the many factors that can also act in this way in packaged water. This is supported by Staples [53], who states that aerobic degradation is the dominant process in the decrease or disappearance of bisphenol A in an aquatic environment. In four weeks, degradation was complete in samples W2 and W6, almost complete in samples W1, W3, W4, W7, W8, and W9, and low in samples W5 and W10. This degradation time is close to that of West et al. [54], who found that under aerobic conditions, BPA degrades for 28 days. Other authors, such as Kang et Kondo [55], who carried out experiments in aerobic and anaerobic conditions with river water spiked with BPA, have concluded that BPA degrades rapidly in the presence of oxygen and that oxidation and/or the effect of anaerobic bacteria have little or virtually no capacity to degrade BPA. This thesis is supported by most authors, who confirm that BPA degrades in the presence of oxygen. Its half-life cycle in the environment is 3 to 5 days in the presence of oxygen. However, it is highly resistant in anoxic aquatic environments, with an assumed half-life of over a year [56]. Microbiology is also a parameter responsible for the elimination of BPA. Microbial analyses revealed the presence of the total germs in all the samples studied. These germs have an optimum growth temperature between 28 and 35 °C, which is close to 39 °C, the highest temperature in this study. The results of these analyses are shown in Table 4. Among these total germs, some are capable of degrading BPA to the point of total or partial mineralisation. This is also the opinion of a European Commission study which, in describing the conditions of the biological degradation of BPA, states that 60% would be mineralised into CO_2_, 20% would constitute the carbon of bacterial cells, and 20% of soluble organic carbon would remain in the media [57].

#### 3.3.4. Chloride and pH Effect on BPA Behaviour

Some authors such as Sakai et al. [58] and Yang et al. [59] have also proposed forms of BPA degradation by specific bacteria, showing that other physico-chemical reactions may be responsible for BPA degradation. This is the case in reactions with chlorides. Thus, by monitoring the presence of chloride ions in the samples for the three study temperatures, it appears that a drop in BPA concentrations is followed by a reduction in chloride concentration in the packaged water. There is then a possible reaction between BPA and chlorides to form new chlorinated substances that no longer have the same physico-chemical characteristics as the initial BPA. This analysis is supported by previous studies which have shown that chlorides react with BPA to give monochloroBPA, dichloroBPA, trichloroBPA, and tétrachloroBPA [60]. Table 5 shows changes in chloride concentrations as a function of time and temperature.

After eight weeks of conditioning, the results show an increased degradation of BPA in all samples except sample W10 at a temperature of 39 °C. This seems to be closely linked to pH, as observed for only the sample at 39 °C with a pH above 8, as shown in Table 6. This suggests that the degradation of BPA is not very efficient at high pH. Chauveheid et al. [60] reported in their study of exposure to BPA and its chlorinated derivatives that degradation of this substance occurs at around pH 7.7 and is clearly ineffective at pH 8.0 and above.

### 3.4. Trace Metals in Packaged Water

All the packaged water carry an insignia printed in ink. The ink products can therefore migrate inside the product. These include trace metals. Cadmium is a micropollutant that can be transferred from the sachet to the water. Cadmium was absent in all samples of the borehole water sources used for the production. Concentrations varied according to the temperature and exposure time of the samples. Table 7 shows cadmium levels in packaged water at different temperatures and as a function of time. Concentrations ranged from 0.0 to 9.7 µg/L. These results show an increase in cadmium concentration in the conditioned water as a function of temperature and duration of storage. These concentrations increase as the exposure time increases and/or the temperature rises.

**Figure 5:**
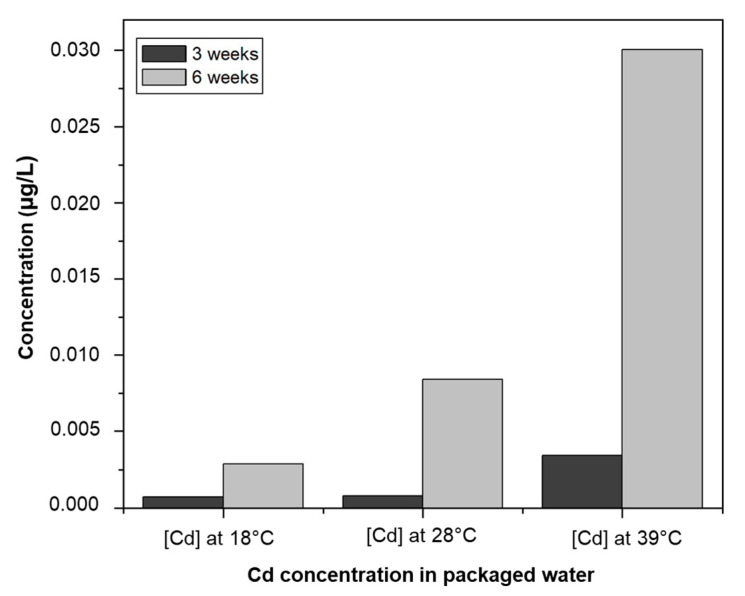
Cd evolution in sachet water as a function of time and temperature.

After six weeks of storage, cadmium was found in all the packaged water studied at 39 °C. On the other hand, the trend in Figure 5 shows that the increase in temperature speeds up the transfer of cadmium to the drinking water. Plastic containers would therefore be responsible for releasing cadmium into packaged water. This migration of cadmium from the sachet to the packaged water is influenced by temperature and contact time. As some sachets have ink marks on the tabs and not on the sachets, we can conclude that the cadmium is not only in the ink, but also in the composition of the sachet.

WHO guidelines for drinking water indicate a limit of 3.0 µg/L cadmium in drinking water. The results of analyses of samples stored for six weeks show that at 18 °C and 29 °C, concentrations vary from 0.0 to 5.1 µg/L, with the exception of W8 at 29 °C which recorded a concentration of 5.1 µg/L, above the recommended standard. At 39 °C, samples W1, W2, W4, W6, and W9, with concentrations of less than 3.0 µg/L, complied with the standard, while samples W3, W5, W7, W8, and W10 had concentrations ranging from 6.6 to 9.7 µg/L, which were two or three times higher than the limit value. Accordingly, depending on the temperature and storage time, some packaged waters may become unfit for consumption because they do not comply with the drinking water standards in force in Burkina Faso.

Table 8 shows the results of iron concentration in the borehole water used as a source for producing the packaged water. Iron was found in all the samples at concentrations ranging from 0.10 to 0.45 mg/L. The concentration for samples F1, F2, F3, F4, F5, F7, and F10 comply with the WHO allowable limit of 0.3 mg/L in drinking water. Samples F6 and F9, with concentrations of 0.45 and 0.40 mg/L, respectively, exceeded the standard limit. This excessive amount of iron is thought to be due to dissolution of the rocks followed by leaching of the soil or cracking of the parent rock [61].

**Figure 6:**
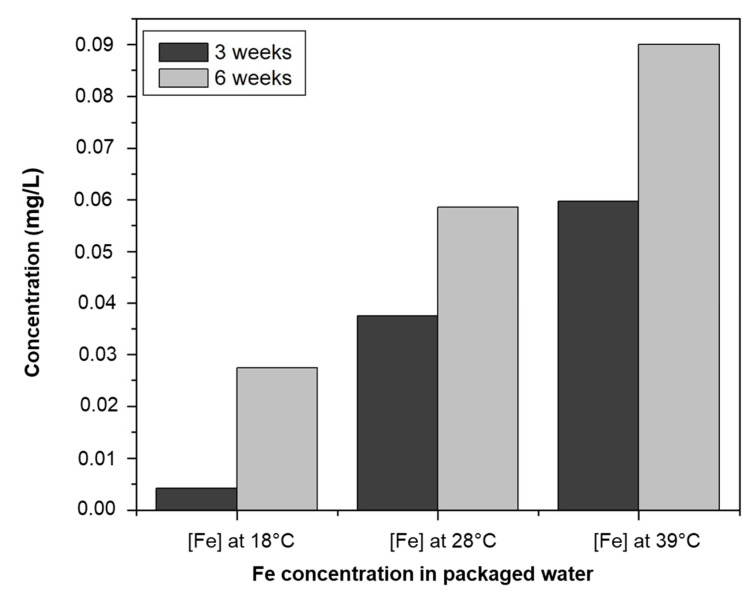
Fe evolution in sachet water as a function of time and temperature.

The impact of environmental conditions such as temperature and storage time on iron transfer from the container to the water consumed was studied (Figure 6). The iron present in the borehole water was found to be completely absent in the packaged water samples W1, W2, W3, W4, W5, and W6, and in small quantities in the packaged water samples W7, W8, W9, and W10 after three weeks and at 18 °C. This can be explained by the treatment method adopted by the packaged water production units. These units sometimes use pre-treatment devices that can retain certain minerals such as iron. As the temperature was increased over time, iron was found in virtually all the samples at increasingly higher concentrations. Iron, like the cadmium present in the sachets, migrates into the packaged water as a function of temperature and time (Table 9). This is why iron concentrations are constantly increasing in packaged water samples. However, according to some authors, iron reacts with BPA in solution, and its concentration must therefore fall in the water [62]. This could be due to the fact that the quantity of iron released is greater than the quantity of iron that reacts with bisphenol A. This always gives an increasing trend that does not reveal this reaction. On the other hand, the absence of or very weak reactions with bisphenol A can be explained by the fact that many substances (oxygen, chloride, etc.) and many bacteria already react with BPA. This gives iron little or no opportunity to react with BPA. In light of Table 7 and Table 9, the migration kinetics of the trace metals is not only a function of the content but also of the alloys between the iron and other trace metals. The degree to which substances migrate into the contents depends not only on the concentrations and chemical characteristics of the compounds, but also on the chemical composition of the plastic [63]. In other words, the presence of certain chemical compounds is likely to create alliances and prevent the migration of other compounds.

## 4. Conclusions

This study assessed the influence of plastic on the quality of packaged water. The survey revealed differences in production conditions depending on the size of the packaged water production structure. Poor storage conditions were found in the places where packaged water is marketed. All the water sources analysed were BPA-free; some contained trace metals, but without exceeding the WHO guidelines for drinking water. The sachets are designed on the basis of BPA to ensure their resistance and transparency, and on the basis of metallic trace elements to accelerate their biodegradability. Under certain physico-chemical and biological conditions, these substances can migrate to the contents (packaged water). During the first thirty days, there was a massive migration of BPA into the water due to the biodegradation of the plastic, which could, in this case, be considered as a source of contamination of substances. After thirty days, BPA gradually degraded and began to disappear after sixty days in some samples. Bottled water contains less BPA when stored at low temperatures at the start of production (before two weeks) and late after production (after two months). BPA is toxic depending on the quantity absorbed and the weight of the consumer. The migration of chemical elements does not depend on the physical nature of the sachets, but rather on the type of chemical compound and storage conditions. Iron and cadmium are also used in the manufacture of sachets and constantly migrate into packaged water due to biodegradation and physico-chemical processes. The geographical location and the presence or absence of a tab on the sachet did not influence the results obtained.

## Figures and Tables

**Table 1:** Physical characteristics of the water sachet and geographical distribution of the samples.

Sample Code	Roughness	Presence of Tongue
W1	Smooth	Yes
W2	Smooth	No
W3	Rough	Yes
W4	Smooth	Yes
W5	Rough	No
W6	Rough	No
W7	Smooth	No
W8	Smooth	No
W9	Rough	No
W10	Smooth	No

**Table 2:** Iron, manganese, and cobalt content given by the XRF tester.

Sample Code	Parameters (mg/L)			Standard Results	Results P/F/X
	Fe	Mn	Co		
W1	33	38	2	Oxo Biodegradable	Validated
W2	24	48	2	Oxo Biodegradable	Validated
W3	52	16	3	Oxo Biodegradable	Undetermined
W4	38	25	3	Oxo Biodegradable	Validated
W5	58	12	2	Oxo Biodegradable	Undetermined
W6	73	13	2	Oxo Biodegradable	Undetermined
W7	81	17	2	Oxo Biodegradable	Validated
W8	75	32	3	Oxo Biodegradable	Validated
W9	45	47	4	Oxo Biodegradable	Validated
W10	70	33	4	Oxo Biodegradable	Validated

**Table 3:** Bisphenol A recovery rates using GC/MS.

Concentrations of Spiked Solutions (mg/L)	Concentrations Detected by GC/MS (mg/L)	Recovery Rate (%)
1.00	1.01	101
5.00	5.20	104
10.00	10.50	105
Average rate		103

**Table 4:** Total germs in packaged water samples.

Samples	W1	W2	W3	W4	W5	W6	W7	W8	W9	W10
Total germs at 37 °C (CFU/L)	440	572	799	404	76	64	900	784	484	816

**Table 5:** Chloride content of samples after 60 days of conditioning.

Exposure Temperature	Chloride Concentration (mg/L)									
	W1	W2	W3	W4	W5	W6	W7	W8	W9	W10
18 °C	1.1	0.8	0.8	0.9	0.8	1.0	0.9	0.8	0.9	0.8
29 °C	0.9	0.8	0.8	0.9	0.7	0.9	0.8	0.8	0.9	0.7
39 °C	0.5	0.6	0.5	0.7	0.6	0.6	0.7	0.7	0.6	0.6

**Table 6:** pH of samples after 8 weeks of storage.

Exposure Temperature	pH Value									
	W1	W2	W3	W4	W5	W6	W7	W8	W9	W10
18 °C	7.3	7.6	6.9	7.2	7.3	7.2	7.2	7.0	7.2	7.5
29 °C	7.5	7.7	7.6	7.3	7.4	7.2	7.6	7.7	7.3	7.8
39 °C	7.6	7.8	7.7	7.4	7.5	7.8	7.7	7.9	7.4	8.1

**Table 7:** Cadmium content in conditioned water samples.

Temperature	Cd Concentration (µg/L) *											Exposure Time
	W1	W2	W3	W4	W5	W6	W7	W8	W9	W10	Average	
18 °C	<DL	<DL	<DL	<DL	<DL	<DL	<DL	<DL	<DL	<DL	<DL	3 weeks
29 °C	<DL	<DL	<DL	<DL	<DL	<DL	<DL	0.9	<DL	<DL	0.1	
39 °C	2.0	1.0	2.5	<DL	<DL	<DL	<DL	5.9	<DL	<DL	1.1	
18 °C	1.0	<DL	<DL	<DL	<DL	<DL	<DL	<DL	<DL	<DL	0.1	6 weeks
29 °C	1.0	<DL	<DL	<DL	<DL	1.0	3.5	5.1	<DL	0.1	1.1	
39 °C	2.0	2.5	6.9	1.0	9.7	2.0	6.6	6.2	1.1	7.2	5.9	

* The admissible limit for Cd in drinking water in accordance with WHO guidelines is 3 µg/L.

**Table 8:** Iron content in borehole water samples.

Water Source	F1	F2	F3	F4	F5	F6	F7	F8	F9	F10
Iron concentration (mg/L)	0.25	0.20	0.15	0.30	0.10	0.45	0.25	0.25	0.40	0.10

**Table 9:** Iron content in conditioned water samples.

Temperature	Fe Concentration (mg/L)											Exposure Time
	W1	W2	W3	W4	W5	W6	W7	W8	W9	W10	Average	
18 °C	<DL	<DL	<DL	<DL	<DL	<DL	0.015	0.017	0.001	0.009	0.004	3 weeks
29 °C	0.030	0.036	0.038	0.003	0.043	0.009	0.069	0.064	0.031	0.054	0.037	
39 °C	0.052	0.039	0.054	0.003	0.082	0.095	0.069	0.081	0.067	0.054	0.059	
18 °C	0.010	0012	<DL	0.008	0.036	0.032	0.059	0.039	0.044	0.034	0.027	6 weeks
29 °C	0.032	0.055	0.069	0.016	0.046	0.051	0.080	0.067	0.083	0.086	0.059	
39 °C	0.076	0.054	0.081	0.100	0.132	0.098	0.086	0.084	0.088	0.102	0.090	

## Data Availability

Not applicable.
